# The Effects of Antimicrobial Peptide Nal-P-113 on Inhibiting Periodontal Pathogens and Improving Periodontal Status

**DOI:** 10.1155/2018/1805793

**Published:** 2018-03-15

**Authors:** Hongyan Wang, Lisi Ai, Yu Zhang, Jyawei Cheng, Huiyuan Yu, Chen Li, Dongmei Zhang, Yaping Pan, Li Lin

**Affiliations:** ^1^Department of Periodontics, School of Stomatology, China Medical University, Shenyang 110002, China; ^2^Department of Periodontics, Jinan Stomatological Hospital, Jinan 250001, China; ^3^Shenyang Dental Hospital, Shenyang 110002, China; ^4^Institute of Biotechnology and Department of Medical Science, National Tsing Hua University, Hsinchu 300, Taiwan

## Abstract

Periodontal disease consists of chronic gingival inflammation characterized by both degradation of the periodontal connective tissue and alveolar bone loss. Drug therapy is used as an auxiliary treatment method in severe chronic periodontitis, aggressive periodontitis, and periodontitis-associated systemic disease. Nal-P-113, a modified antimicrobial peptide, specifically replaces the histidine residues of P-113 with the bulky amino acid *β*-naphthylalanine, and our previous studies have verified that this novel peptide is not toxic to the human body within a certain concentration range. The objective of the present study was to evaluate the effect of Nal-P-113 on periodontal pathogens and periodontal status in clinical studies. In a split-mouth clinical trial, the pocket depth and bleeding index values tended to decrease in the experimental group compared with those in the control group. SEM results verified that Nal-P-113 restrained the maturation of plaque. Based on real-time polymerase chain reaction, the levels of* Fusobacterium nucleatum*,* Streptococcus gordonii*,* Treponema denticola, *and* Porphyromonas gingivalis* in subgingival plaque were decreased when the subjects were given Nal-P-113. Bacterial growth curve analysis and a biofilm susceptibility assay verified that Nal-P-113 at a concentration of 20 *μ*g/mL restrained the growth of* S. gordonii*,* F. nucleatum,* and* P. gingivalis* and biofilm formation. Therefore, Nal-P-113 effectively reduces periodontal pathogens and ameliorates periodontal status.

## 1. Introduction

Periodontal disease consists of chronic gingival inflammation caused by multiple microbes [1]. Periodontitis, a high-incidence disease and the key causation of tooth loss worldwide [2], is characterized by both degradation of the periodontal connective tissue and alveolar bone loss. In the development of periodontitis, plaque accumulation results in periodontal tissue inflammation, the formation of a periodontal pocket, and alveolar bone damage [3]. As shown in previous studies, many periodontal microbiota, including both Gram-negative and Gram-positive bacteria, are closely associated with chronic periodontitis [4–6]. Among these bacteria,* Tannerella forsythia, Fusobacterium nucleatum,* and* Porphyromonas gingivalis* are considered closely linked with the occurrence of periodontitis [7]. Furthermore,* Prevotella intermedia*,* Treponema denticola*,* Actinomyces viscosus,* and* Streptococcus gordonii* may play a keystone role in dental plaque biofilm formation and in accelerating chronic periodontitis progression [8]. Conventional periodontal treatments include scaling and root planing to eliminate plaque and other pathogenic factors. However, drug therapy is used as an auxiliary treatment method in severe chronic periodontitis, aggressive periodontitis, and periodontitis-associated systemic disease [9–11]. Antibiotics can result in many side effects, such as antimicrobial resistance, dysbacteriosis, diarrhoea, pigmentation, and vomiting [12, 13]. Moreover, the therapeutic effect of antibiotics is weakened once the bacteria form a biofilm. Therefore, a new type of biological agent is urgently needed to replace antibiotics.

Cationic antimicrobial peptides (CAMPs), which have been isolated from plants, animals, bacteria, and fungi, are important elements of host innate immunity [14].* In vivo*, CAMPs can defend against invading pathogens [15], such as Gram-negative and Gram-positive bacteria, yeast, viruses, and fungi [16]. The mechanism underlying the antimicrobial activity of CAMPs is related to disruption of the plasma membrane, leading to target cell lysis [6]. In particular, CAMPs have been reported to reduce the growth of periodontal pathogens, including* P. gingivalis*,* in vitro *[17, 18]. However, most CAMPs exhibit reduced antibacterial activity once they are exposed to physiological fluids such as blood, plasma, saliva, serum, or sputum [19].

In humans, the parotid and submandibular-sublingual glands secrete histidine-rich peptides called histatins [5], which are small, cationic peptides. According to previous studies, histatins alter the permeability of cell membranes [20, 21], and these novel peptides are thus expected to have low toxicity and the potential for preventive and therapeutic effects. In one study on beagles, histatins markedly inhibited the formation of plaque and gingival inflammation [22]. P-113 has been identified as the minimum segment of histatin 5 that maintains the antimicrobial activity of the parent compound [23]. In Nal-P-113, a variant of P-113, the histidine residues in 4, 5, and 12 loci are replaced by *β*-naphthylalanine. Our previous studies have verified that this novel peptide is not toxic to the human body within a certain concentration range [24]. In addition, Nal-P-113 has a stronger bactericidal ability and greater stability in blood, saliva, and salt solution than P-113 does [25], indicating its potential applicability in the clinic.

In the present study, two double-blinded, randomized clinical studies were designed to evaluate the ability of Nal-P-113 in gelatine form to control subgingival plaque and improve periodontal status. In planktonic and biofilm state, the antimicrobial ability of Nal-P-113 was also* in vitro* detected.

## 2. Materials and Methods

### 2.1. Design and Study Population

Double-blinded, randomized clinical studies of Nal-P-113 were conducted. In particular, a split-mouth clinical trial was conducted from August 2014 to January 2015. In total, 37 patients with moderate or severe chronic periodontitis were enrolled in the study from the Department of Periodontics, School of Stomatology, China Medical University (18 females and 19 males, aged between 21 and 55 years, mean age 35.78 ± 10.70 years). The patients were recruited randomly. None of the participants had a habit of unilateral mastication. All patients provided written informed consent for this test. The study was in accordance with the Declaration of Helsinki and was ratified by the Ethics Committee of China Medical University for Clinical Research.

### 2.2. Inclusion and Exclusion Criteria

The chronic periodontitis patients were diagnosed with a pocket depth (PD) ≥ 4 mm and with alveolar bone loss affecting more than one-third of tooth root based on radiographic evidence. In the past three months, the patients had not received antibiotics. The patients also did not receive any periodontal intervention before the baseline examination. If they had any systemic disease known to have an impact on periodontal health or if they were pregnant or lactating, they would be excluded. Patients were also excluded if they were using any mouth rinse or if they were smokers. Moreover, four patients were excluded for using antibiotics during the trial.

### 2.3. Clinical Treatment Method

 All patients first had an intraoral check via Florida Probe (FP32, Version9, USA), and sampling sites on two contralateral teeth with similar conditions were selected. The 74 teeth in the 37 patients were randomly divided into two treatment groups using the split-mouth technique: an experimental group (37 teeth) and a control group (37 teeth). Antimicrobial peptide Nal-P-113 (20 *μ*g/mL) or placebo was then injected into the pocket of the selected teeth in a random and double-blinded manner. The teeth were again treated with Nal-P-113 or placebo in gelatine form at a follow-up visit 3 days after the initial treatment. The selected teeth were then sampled seven days later owing to the fast efficacy of Nal-P-113. After the experiment, all teeth received nonsurgical periodontal therapy, including oral supragingival scaling, subgingival scaling, root planing, and oral hygiene instruction. At baseline (0 days) and on the 7th day, clinical plaque samples were collected, and all parameters were measured.

Ten patients with chronic periodontitis resulting in loose teeth that had to be removed were chosen from among the 37 patients. The loose teeth from 5 patients were applied with Nal-P-113 in gelatine (20 *μ*g/mL), and the other 5 teeth were administered placebo. On the 7th day, all of the loose teeth were pulled for SEM analysis.

### 2.4. Clinical Parameter Detection

The mean values of clinical indexes, including periodontal depth (PD), clinical attachment level (CAL), and bleeding index (BI), were checked at six sites per tooth and recorded before and seven days after administration of medication. This clinical trial has been registered on the Chinese clinical trial website (No. ChiCTR-OIC-16010250).

Correlation coefficients were used to determine interrater and intrarater reliability. Percentage agreement between assessors and the *κ* coefficient with a 95% confidence interval (CI) were used. In our study, the intraexaminer weighted *κ* values (±1 mm) ranged from 0.88 to 0.91, and interexaminer reliability yielded values ranging from 0.77 to 0.80.  Table 1 shows the baseline demographic and clinical characteristics for each group.

### 2.5. SEM Analysis

The removed teeth were fixed with 2.5% glutaraldehyde (BioChemika, Fluka Company, USA), washed glutaraldehyde out by phosphate-buffered saline gently and gradient dehydration with ethanol. Images at 20,000x magnification were gained using scanning electron microscope (Inspect F50, FEI Company, USA).

### 2.6. Sample Collection and Preservation

A sterile paper point was inserted into the bottom of periodontal pocket and left there for 30 s to collect subgingival plaque at each of the selected sites baseline and 7 days after the medication was administered. All of the samples were collected in Eppendorf tubes and stored at −80°C.

### 2.7. Real-Time Polymerase Chain Reaction (PCR)

A Genomic DNA Preparation Kit was used to extract DNA from the subgingival dental plaque.* P. gingivalis*,* T. forsythia*,* F. nucleatum*,* S. gordonii*,* A. viscosus*,* P. intermedia,* and* T. denticola* were detected before and 7 days after administration of the medication. Primer pairs were designed by Primer Premier 5.0 software and BLAST searches for the 16S DNA gene against the GenBank database  (https://www.ncbi.nlm.nih.gov). The universal primer pairs and the primer pairs used for the specific bacteria were as follows:

Universal-F: TGGAGCATGTGGTTTAATTCGA

Universal-R: TGCGGGACTTAACCCAACA

Pg-F: CATAGATATCACGAGGAACTCCGATT

Pg-R: AAACTGTTAGCAACTACCGATGTGG

Tf-F: CGTTTCCGAAGAGTATAACCACA

Tf-R: CATGCAGCTTGATATTCTGAGG

Fn-F: TGCGATAAGCCTAGATAAGTTGCA

Fn-R: CTTAATAGATTGCTCCATTCGGAAA

Pi-F: CGGTCTGTTAAGCGTGTTGTG

Pi-R: CACCATGAATTCCGCATACG

Td-F: CCGAATGTGCTCATTTACATAAAGGT

Td-R: GATACCCATCGTTGCCTTGGT

Sg-F: AGCGCAGGCGGTTAGATAAG

Sg-R: TGCTTAATGCGTTAGCTGCG

Av-F: GGTTCTGGATGAGTGGCGAA

Av-R: TAGCCACACTTTCATGCGGT

QIAamp DNA Mini Kits (Qiagen, UK) were used to extract DNA according to the operating instructions. A quantitative PCR machine (Applied Biosystems 7500, USA) was employed for real-time PCR amplification. The fluorescent system included SYBR Green reaction mixtures consisting of 10 *μ*L QuantiTect SYBR Green I Master Mix (Qiagen, Germany), 2 *μ*L template DNA, and nuclease-free water added to a final volume of 20 *μ*L. The thermocycling programme conditions included an initial incubation at 95°C for 30 s, followed by 40 cycles of two-step PCR at 95°C for 5 s and 58°C for 34 s, then 95°C for 15 s, 60°C for 60 s, and 95°C for 15 s. Universal 16S rDNA expression was calculated as the expression level relative to the respective control. The data were calculated by ABI 7500 soft version 2.0.5. Relative level was quantified with the 2−^ΔΔ^CT method [26].

### 2.8. Bacterial Strains

*F. nucleatum* ATCC25586 and* P. gingivalis* W83 were grown anaerobically (80% N_2_, 10% CO_2_ and 10% H_2_) in brain-heart infusion agar plate added with 1 *μ*g/mL menadione, 10 *μ*g/mL hemin acquired from Sigma, and 5% sheep blood.* S. gordonii* Challis CH1 was grown on a BHI agar plate aerobically [24].

### 2.9. Bacterial Growth Curve

The optical density at 600 nm of* S. gordonii*,* F. nucleatum,* and* P. gingivalis *was adjusted to 0.06–0.07 using BHI broth. The concentration of 20 *μ*g/mL Nal-P-113 was then added to the respective bacterial cultures. Cultures without an antibacterial agent were considered as control group. The plates were incubated for 48 h continuously, a microplate reader was used to analyse the optical density at 600 nm. The tests were performed more than three times.

### 2.10. Biofilm Susceptibility Assay

The effect on biofilm formation by antibacterial agent was detected by the microdilution method [27]. For this purpose, cell suspensions of* S. gordonii* Challis CH1,* F. nucleatum *ATCC25586, and* P. gingivalis* W83 were diluted to 5 × 10^5^ CFU/mL, and 20 *μ*g/mL Nal-P-113 was added to cell suspensions. 95% methanol was used to fix formed biofilms and 0.5% (w/v) crystal violet was applied to stain, after which the optical density at 590 nm was analysed using a microplate reader. The tests were performed more than three times.

### 2.11. Statistical Analysis

The sample size was estimated using a power calculator, assuming that a confidence level of 95%, a 20% reduction in the level of the parameter, and a 10% standard deviation were clinically relevant. A sample size of 30 subjects was required for the split-mouth clinical trial; a power (1−*β*) of > 0.80 was computed for the two-sided null hypothesis.

All statistical analyses were performed with SPSS 13.0, with a significance level of *α* = 0.0500. The normality test was applied to all measured data, but as the data did not accord with a normal distribution, a Wilcoxon signed-rank test was applied to analyse.

## 3. Results

### 3.1. Evaluation of Clinical Parameters

Prior to application of the antimicrobial peptide Nal-P-113 and placebo, no significant difference in PD, CAL, or BI was observed between the experimental and the control groups (*P* > 0.05). Seven days after administration of the medication, the PD decreased for 22 of 33 teeth (4 patients were excluded for using antibiotics during the trial), and the BI values of 26 of the 33 teeth were reduced in the experimental group. The PD and BI values in the experimental group significantly decreased compared to those in the control group (*P* < 0.05). In contrast, no difference between the control group and the experimental group was observed for the CAL (*P* > 0.05, Table 2).

### 3.2. SEM Analysis of Subgingival Plaque

**Figure 1:**
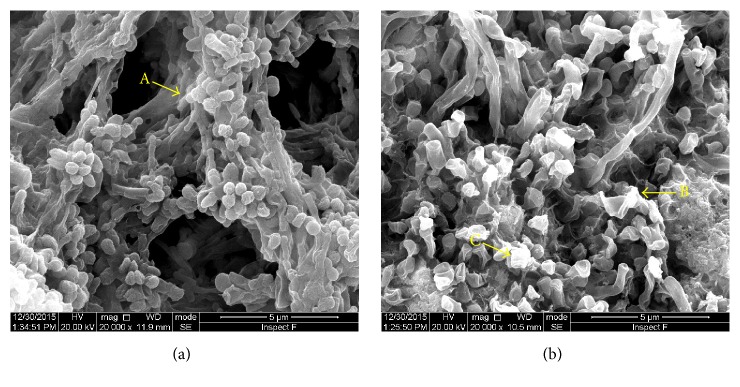
SEM analysis of subgingival plaque structure. (a) The structure of the subgingival plaque from the control group was tight, and the organic substrate comprised the skeleton. A: organic substrate. (b) The bacteria in the subgingival plaque from the experimental group were markedly shrunken. B, C: shrunken bacterial structure.

The adhesive subgingival plaque was mainly composed of* cocci*,* bacilli,* and* filamentous* bacteria as well as short Gram-negative* bacilli* and Spirochaetes. The structure of the subgingival plaque from the control group was tight, and an organic substrate comprised the skeleton (Figure 1(a)). The* cocci*,* bacilli,* and* filamentous* bacteria on the surface of the subgingival plaque from the experimental group were notably shrunken, and the amount of organic substrate was decreased; in addition, the plaque structure had collapsed (Figure 1(b)).

### 3.3. Real-Time PCR

Real-time PCR was used to detect changes in* P. gingivalis*,* T. forsythia*,* P. intermedia*,* T. denticola*,* F. nucleatum*,* S. gordonii,* and* A. viscosus* in dental plaque at baseline and the 7th day. The differences in the levels of* P. gingivalis*,* T. denticola, F. nucleatum,* and* S. gordonii* that were observed in the experimental group compared with the control group were statistically significant (*P* < 0.05, Table 3).

### 3.4. Bacterial Growth Curve

**Figure 2:**
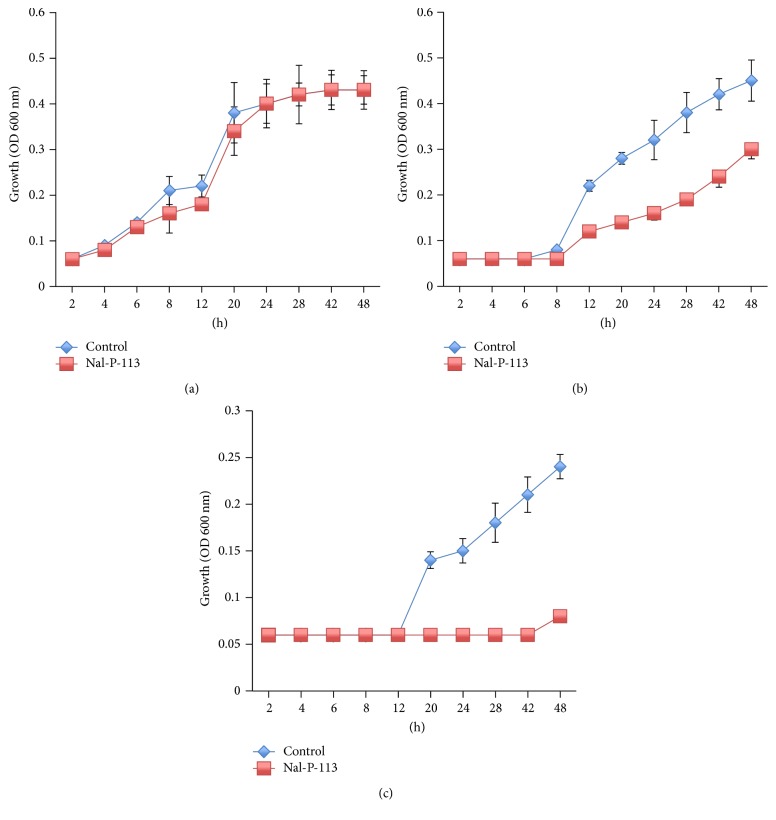
Growth-inhibitory effect of Nal-P-113 (20 *μ*g/mL) on* S. gordonii *Challis CH1,* F. nucleatum* ATCC 25586 and* P. gingivalis *W83. (a)* S. gordonii *Challis CH1; (b)* F. nucleatum* ATCC 25586; (c)* P. gingivalis *W83. The data shown here are the mean* ±* SD from three independent cultures.

Next, we detected the antimicrobial activity of Nal-P-113* in vitro*. Nal-P-113 was added to the growing bacterial strains at a concentration of 20 *μ*g/mL. The data revealed that Nal-P-113 postponed* F. nucleatum* and* P. gingivalis* entry into the exponential growth phase, thus reducing the number of bacteria. For* S. gordonii*, at the similar time point, 20 *μ*g/mL Nal-P-113 slightly reduced the total bacterial amount (Figure 2).

### 3.5. Biofilm Susceptibility Assay

**Figure 3:**
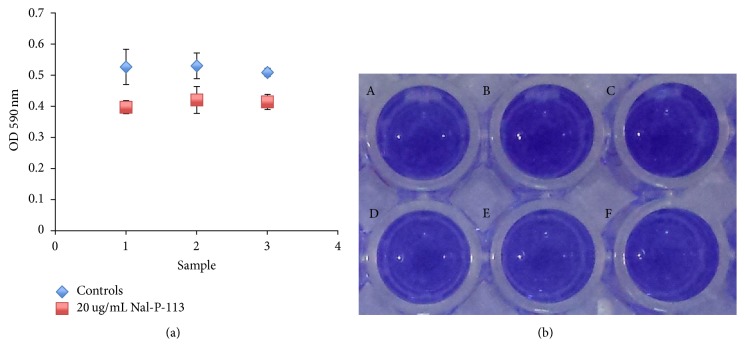
Nal-P-113 (20 *μ*g/mL) inhibited* S. gordonii *Challis CH1,* F. nucleatum* ATCC 25586 and* P. gingivalis *W83 biofilm formation. (1)* S. gordonii *Challis CH1; (2)* F. nucleatum* ATCC 25586; (3)* P. gingivalis *W83.

On the tooth surface, periodontal pathogens exist in the form of dental biofilms. So we examined the ability of Nal-P-113 on inhibiting* S. gordonii *Challis CH1,* F. nucleatum* ATCC25586, and* P. gingivalis* W83 biofilm formation* in vitro*. At a concentration of 20 *μ*g/mL, Nal-P-113 inhibited the above bacterial biofilm formation as expected (shown in Figure 3, *P* < 0.05).

## 4. Discussion

Conventional antibiotics are used excessively and inappropriately in the clinic. Consequently, the resistance of pathogens has increased, the effect of antibiotics has weakened, and the number of multidrug-resistant pathogens that are resistant to most antibiotics has increased [28]. Researchers must solve this problem by developing new compounds. Among new biomedical alternatives, CAMPs are considered candidates with great promise [29–31]. CAMPs specifically comprise avaried group of small proteins which exert various biological functions, like antimicrobial activity, anticancer activity, antihypertensive activity, anti-inflammatory activity, and so forth [30]. Histatins are one family of CAMPs that have been revealed to protect the healthy host from oral bacteria such as* P. gingivalis *[32, 33]. In experiments using beagles, histatins were also shown to suppress plaque formation and gingival inflammation [22]. However, CAMPs extracted from a living organism cannot be applied clinically because they are degraded in the presence of high salt concentrations. Indeed, although most CAMPs have high activity under physiological conditions, they exhibit greatly reduced antibacterial activity upon exposure to physiological fluids such as blood, plasma, saliva, serum, or sputum [34–36]. Nal-P-113 contains 12-amino-acid fragment from saliva protein histatin 5, with histidine replaced with *β*-naphthylalanine at positions 4, 5, and 12 to increase the structural stability, screw stability, and hydrophobicity of the parent compound, namely, the CAMP P-113 [24]. Based on our previous research, Nal-P-113 can seriously perforate the cytoplasmic membrane and cause the cytoskeleton to completely collapse, and the bactericidal function of Nal-P-113 is stronger and faster than that of metronidazole or penicillin* in vitro *[24]. Accordingly, we designed a short-term study to assess the effect of this drug.

In* in vivo *environments, many bacteria will form a dental biofilm. Antibiotics and CAMPs cannot easily penetrate periodontal biofilms. In the present study, we designed a split-mouth clinical trial to evaluate the clinical efficacy of Nal-P-113, including its ability to control periodontal pathogens* in vivo*. To eliminate the impact of mechanical removal on the periodontal clinical index, to clearly reveal the effects of the drug, and to explicitly determine the change in subgingival bacteria, Nal-P-113 was administered before scaling and root planing. After injection of the antimicrobial peptide gel or placebo into the pocket of the selected teeth for 7 days, the PD (*P* = 0.039) and BI (*P* = 0.012) values of the experimental group showed a decreasing trend compared with those of the control group. Based on this result, the antimicrobial peptide Nal-P-113 entered the subgingival dental plaque and alleviated gingival inflammation in the human oral environment. Regarding the CAL, the value in the experimental group did not show an obvious difference compared with that in the control group (*P* = 0.386). Our data thus confirmed that Nal-P-113 effectively controlled subgingival plaque and improved periodontal status* in vivo*.

The formation and accumulation of dental plaque biofilm are the direct cause of periodontal diseases [37]. Saliva proteins and glycoproteins first adsorb to the tooth surface and form the acquired pellicle, after which oral bacteria colonize this pellicle, and the plaque biofilm matures [38, 39]. As the mature plaque biofilm is an integral micropopulation, it is difficult to clear, resulting in antibiotic avoidance. Periodontal treatments are even more complex when bacteria form biofilms. Thus, strategies that prevent plaque biofilm formation and destroy the biofilm structure are necessary for periodontal treatment. According to our SEM results, the bacteria on the surface of the subgingival plaque from the experimental group were notably shrunken, and the organic substrate was decreased. In addition, the plaque structure had collapsed. Based on these results, Nal-P-113 restrained bacterial biofilms formation or maturation and destroyed the skeleton of the subgingival plaque, confirming the efficacy of Nal-P-113 on morphology. The* in vitro* biofilm susceptibility assay also confirmed that Nal-P-113 at a concentration of 20 *μ*g/mL could reduce* S. gordonii*,* F. nucleatum*, and* P. gingivalis* biofilm formation.

*P. gingivalis, T. forsythia, *and* F. nucleatum *are closely related to periodontitis, and* S. gordonii*,* T. denticola, *and* P. intermedia *are common anaerobic bacteria that are also related to periodontitis caused by dental plaque. Accordingly, we detected these bacterial levels in subgingival plaque. The populations of* P. gingivalis*,* T. denticola*,* F. nucleatum, *and* S. gordonii* were decreased in most samples following the use of the Nal-P-113 gelatine, and the levels were significantly decreased (*F. nucleatum*: *P* = 0.021,* S. gordonii*: *P* = 0.017,* P. gingivalis*: *P* = 0.044, and* T. denticola*: *P* = 0.045) compared with those in the control group. We hypothesized that one of the reasons was that Nal-P-113 restrained the growth of the above-mentioned bacteria. Therefore, the growth curves of* S. gordonii*,* F. nucleatum, *and* P. gingivalis* strains were examined following 20 *μ*g/mL Nal-P-113 application. The results showed that at a concentration of 20 *μ*g/mL, Nal-P-113 reduced the number of* F. nucleatum* or* P. gingivalis* bacteria by postponing entry into the exponential growth phase. In addition, Nal-P-113 slightly restrained the growth of* S. gordonii* at the same time point. Meantime, in the clinical trial, though* T. forsythia* and* P. intermedia *populations were reduced in the experimental group, no obvious or significant differences were detected between the experimental and the control groups (*P* > 0.05), possibly due to the low concentration of Nal-P-113 used.

## 5. Conclusions

This research verified that Nal-P-113 might be effectively applied to clinical practice as a biological agent. It improved periodontal clinical status by inhibiting periodontal pathogens growth and controlling dental biofilms formation. Nal-P-113 may be an auxiliary means to prevent or cure periodontitis in the future.

## Figures and Tables

**Table 1:** Baseline demographic and clinical characteristics.

Group	Number	Age/years	Sex		PD		CAL		BI	
		(mean ± SD)			(mean ± SD)		(mean ± SD)		(mean ± SD)	
			male	female	experimental	control	experimental	control	experimental	control
Split-mouth clinical trial	37	35.78 ± 10.70	19	18	5.45 ± 1.58	5.58 ± 1.50	3.12 ± 1.29	3.15 ± 1.25	3.00 ± 0.75	2.97 ± 0.76
*P*					0.33		0.71		0.41	

**Table 2:** Comparison of the different values of clinical indicators between the two groups in the split-mouth study.

Index	Experimental group				Control group				*P*
(mm)	mean	median quality	upper quality	lower quality	mean	median quality	upper quality	lower quality	
PD	0.76	1.00	.00	1.00	0.49	.00	.00	1.00	0.039^*∗*^
CAL	0.21	.00	.00	0.25	0.14	.00	.00	.00	0.386^*∗*^
BI	1.12	1.00	1.00	2.00	0.73	1.00	.00	1.00	0.012^*∗*^

*∗* indicates *P* < 0.05 compared with the control group.

**Table 3:** Comparison of the different subgingival plaque values between the two groups in the split-mouth study.

Bacteria	Group	Median	Upper quartile	Lower quartile	*P*
*Pg*	experimental	.0956	.0011	.2487	0.044^*∗*^
	control	.0009	−.0201	.1704	
*Tf*	experimental	.0000	−.0021	.0109	0.339
	control	.0001	−.0009	.0162	
*Pi*	experimental	.0097	−.0090	.0358	0.376
	control	−.0001	−.0432	.0149	
*Td*	experimental	.0094	−.0021	.0165	0.045^*∗*^
	control	.0028	−.0082	.0150	
*Fn*	experimental	0.01088	0.02942	0.00175	0.021^*∗*^
	control	0.01822	0.04239	0.00299	
*Sg*	experimental	.01304	0.1740	0.0781	0.017^*∗*^
	control	0.1876	0.2362	0.0937	
*Av*	experimental	0.0001911	0.0005142	0.000907	0.133
	control	0.0002786	0.0006597	0.000977	

*∗* indicates *P* < 0.05 compared with the control group.

## Supplementary Materials

Supplementary Materials CONSORT 2010 checklist of information to include when reporting a randomized trial.
